# Possible Prognostic Role of BMI Before Chemotherapy in the Outcomes of Women with Ovarian Cancer

**DOI:** 10.3390/nu17030556

**Published:** 2025-01-31

**Authors:** Stavroula A. Paschou, Angeliki Andrikopoulou, Nikoletta Mili, Anna Svarna, Maria Kaparelou, Katerina Stefanaki, Nikolaos Dedes, Efstathia Liatsou, Nikolaos Thomakos, Dimitrios Haidopoulos, Theodora Psaltopoulou, Efstathios Kastritis, Flora Zagouri, Meletios-Athanasios Dimopoulos, Michalis Liontos

**Affiliations:** 1 Endocrine Unit and Diabetes Center, Department of Clinical Therapeutics, Alexandra Hospital, School of Medicine, National and Kapodistrian University of Athens, 80 Vasilisis Sophias, 11528 Athens, Greece; spaschou@med.uoa.gr (S.A.P.); k.stefanaki@hotmail.gr (K.S.); 2 Hematology and Oncology Unit, Department of Clinical Therapeutics, Alexandra Hospital, School of Medicine, National and Kapodistrian University of Athens, 11528 Athens, Greece; aggandrikop@med.uoa.gr (A.A.); anna.svarna@hotmail.com (A.S.); kapareloum@gmail.com (M.K.); dedes.nik.95@gmail.com (N.D.); fayliatsou1@gmail.com (E.L.); tpsaltop@med.uoa.gr (T.P.); ekastritis@med.uoa.gr (E.K.); fzagouri@med.uoa.gr (F.Z.); mdimop@med.uoa.gr (M.-A.D.); 3 2nd Department of Obstetrics and Gynaecology, Aretaieio Hospital, School of Medicine, National and Kapodistrian University of Athens, 11528 Athens, Greece; nikoletta.mili@gmail.com; 4 1st Department of Obstetrics and Gynaecology, Alexandra Hospital, School of Medicine, National and Kapodistrian University of Athens, 11528 Athens, Greece; nthomakos@med.uoa.gr (N.T.); dchaidop@med.uoa.gr (D.H.)

**Keywords:** ovarian cancer, body mass index, BMI, obesity, chemotherapy, survival

## Abstract

Background/Objectives: Survival rates for ovarian cancer remain distressingly low. Despite established prognostic factors, the need to identify modifiable parameters to influence survival outcomes is imperative. Overweight and obesity, both prevalent conditions, have been implicated in cancer development and potentially poor survival. However, conflicting data on the associations of body mass index (BMI) with progression-free survival (PFS) and overall survival (OS) in ovarian cancer patients necessitate further exploration. This study aims to investigate the prognostic role of BMI before chemotherapy in women with ovarian cancer, specifically focusing on PFS and OS. Methods: A retrospective analysis encompassed 1,136 patients diagnosed with ovarian carcinomas between 1995 and 2018. Patients were categorized based on BMI at presentation, and a comprehensive examination of clinicopathological, treatment, and survival data was conducted. Results: In the patient population, normal weight patients (BMI < 25 kg/m^2^) demonstrated a median PFS of 12.8 months (95% CI 11.7–13.9 months), while overweight/obese patients (BMI ≥ 25 kg/m^2^) exhibited a significantly longer median PFS of 14.9 months (95% CI 13.6–16.4 months, *P* = 0.006). No statistically significant difference was noted in median OS between the two BMI groups. Subgroup analysis for different histological subtypes revealed a statistically significant benefit for overweight and obese patients with serous and endometrioid histology (mPFS 12.9 months, 95% CI 11.7–14.0 vs. 15.6 months, 95% CI 13.9–17.3, *P* = 0.012 and 14.6 months 95% CI 13.7–15.5 vs. 25.6 months, 95% CI 9.5–41.7, *P* = 0.031, respectively). Additionally, BMI ≥ 25 kg/m^2^ demonstrated a significant advantage in advanced-stage disease. Conclusions: The study underscores the intricate association between BMI and ovarian cancer prognosis. While a statistically significant difference in progression-free survival was noted between normal weight and overweight/obese patients, with the latter group experiencing a survival benefit, no such difference was observed in overall survival.

## 1. Introduction

Survival of patients with ovarian cancer is poor. Less of 50% of women will be alive 5 years after diagnosis, rendering ovarian cancer one of the most lethal gynecologic malignancies [1,2]. The key prognostic factors, such as age, grade of the tumor and stage of the disease, are not modifiable at diagnosis. The understanding of potentially modifiable parameters could probably influence survival and could reduce a woman’s risk of disease progression or cancer recurrence.

Overweight and obesity have become epidemic conditions currently, affecting around 40% and 15% of the general adult population, respectively [3]. This state of excessive fat accumulation, mainly expressed in everyday clinical practice as body mass index (BMI, kg/m^2^), is associated with the development of various types of cancer and probably poor survival outcomes [4]. However, data on the associations of BMI with progression-free survival (PFS) and overall survival (OS) in patients with ovarian cancer remain conflicting. Results from the Ovarian Cancer Association Consortium (OCAC) suggest a shorter overall survival (OS) and progression-free survival (PFS) in obese patients compared to those with normal weight [5]. Studies on pre-diagnosis obesity showed mixed results, with some associating BMI ≥ 25 kg/m^2^ with increased mortality [6,7,8,9,10,11,12]. While initial findings linked higher BMI at treatment initiation to shorter OS and PFS, subsequent studies found no significant association [13,14,15]. In contrast, other studies showed a trend towards improved survival in ovarian cancer patients with BMI ≥ 25 [16,17], while BMI reduction during or after chemotherapy was linked to poorer outcomes [18,19,20].

As this association needs further investigation, we aimed to investigate the prognostic role of BMI before chemotherapy in the outcomes of women with ovarian cancer, focusing on progression-free survival (PFS) and overall survival (OS).

## 2. Materials and Methods

### 2.1. Study Design

This is a single institution retrospective study. Patients with histologically confirmed ovarian cancer, treated in the Department of Clinical Therapeutics, Alexandra Hospital, School of Medicine, National and Kapodistrian University of Athens from 1995 to 2018 were included in the analysis. All patients had given their written consent for the use of their medical data. The study was granted approval by Institutional Review Board and was conducted according to the Declaration of Helsinki.

All patients were divided in groups according to their BMI at the start of chemotherapy. Initially, a comparison between those with BMI < 25 kg/m^2^ and those with BMI ≥ 25 kg/m^2^ was performed. We further investigated whether there was a linear trend between increased BMI and survival in the cohort. Thus, we categorized patients as normal weight (BMI 15–24.99 kg/m^2^), overweight (BMI 25–29.99 kg/m^2^), obese class I (BMI 30–34.99 kg/m^2^) and obese class II (BMI > 35 kg/m^2^).

Clinicopathological, treatment and survival data were collected from patients’ records. More specifically, demographical data including patients’ date of birth, age at diagnosis and date of first disease progression and/or death were collected. The type of surgery included primary or interval debulking and surgery outcome was defined as optimal or suboptimal. Tumor staging was performed in accordance with the FIGO staging system for ovarian cancer. Data regarding chemotherapy regimens, namely treatment with a combination of paclitaxel and carboplatin or carboplatin alone were also collected. Patients’ performance status was measured according to ECOG Scale Performance Status.

### 2.2. Statistical Analysis

All data were coded and analyzed using a specifically designed database of the SPSS statistical package (SPSS Inc., Armonk, NY, USA) version 24. The Kolmogorov–Smirnov test was used to assess the normality of the data. The outcome of the debulking surgery was classified as optimal (absence of residual disease or residual disease below 1 cm) or suboptimal (residual disease more than 1 cm). OS was defined as the time between the date of diagnosis and the date of death from any cause. PFS was defined as the time between the date of diagnosis and the date of progression. Alive patients were censored at the date of last contact.

Kaplan–Meier estimates were used to describe and visualize the effect of categorical variables on OS and PFS. Survival analysis was calculated by Kaplan–Meier curves and survival differences between groups were compared using the log-rank test. The estimation of the prognostic value of several variables with patients’ survival was performed by Cox regression models. Multivariate Cox regression analysis was used to estimate the independent predictive value of the various factors in patients’ survival. Known prognostic factors for ovarian carcinoma (i.e., age, stage, histology, quality of debulking surgery, ECOG performance status) as well as BMI groups were included in the multivariate analysis. All statistical correlations were considered significant at the level of *P* < 0.05.

## 3. Results

### 3.1. Baseline Characteristics

Between 1995 and 2018, 1,136 patients diagnosed with ovarian carcinomas that were treated in our institution and were included in the analysis. The population consisted mainly of advanced-stage patients (77.3%), most of whom had serous histology (61/6%), while endometrioid carcinomas were the second most frequently encountered histology (12.1%). The vast majority of the patients were subjected to primary debulking surgery (86.1%) but there was a significant percentage of patients whose surgical outcome was suboptimal (46.1%). Among them, 514 patients (45.2%) had BMI < 25 kg/m^2^ at diagnosis while 618 patients (54.4%) were overweight or obese (BMI ≥ 25 kg/m^2^). In the total population, median age was 60.4 years (25th–75th percentile 51.2 to 68.6 years), median BMI was 25.5 (25th–75th percentile 22.6 to 29.3 kg/m^2^) and median body surface area (BSA) was 1.65 m^2^ (25th–75th percentile 1.55 to 1.76 m^2^). Baseline characteristics of the entire population as well as of patients with BMI < 25 or ≥25 kg/m^2^ are displayed in Table 1. Except for the stage, there were no statistically significant differences between the two groups in all of the examined clinicopathological characteristics that were known to have prognostic significance in ovarian carcinomas. More specifically, a higher percentage of patients with BMI ≥ 25 were diagnosed with early ovarian cancer in comparison to their normal weight counterparts (*P* = 0.023) at baseline. Clinicopathological characteristics of the subpopulation of patients according to BMI groups are displayed in Supplementary Table S1.

### 3.2. Survival

**Figure 1:**
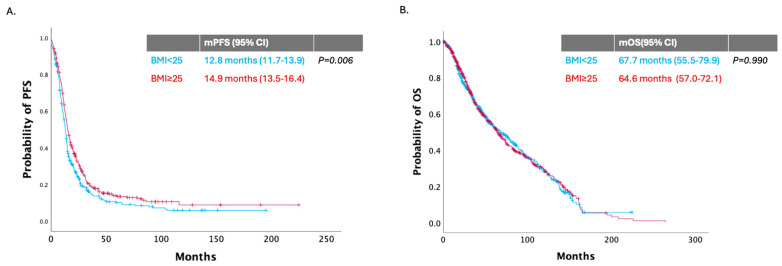
Kaplan–Meier curves of: (**A**) PFS, and (**B**) OS according to BMI in the overall population with BMI ≥ 25 kg/m^2^ (red line) and BMI < 25 kg/m^2^ (blue line).

In the whole population, there was a statistically significant difference in median PFS (mPFS) between normal weight (BMI < 25 kg/m^2^) and overweight/obese patients (BMI ≥ 25 kg/m^2^). More specifically, patients with BMI < 25 kg/m^2^ had mPFS of 12.8 months (95% CI 11.7–13.9 months), while in those with BMI ≥ 25 kg/m^2^, mPFS was 14.9 months (95% CI 13.6–16.4 months, *P* = 0.006). On the contrary, no statistical difference was noted in median OS (mOS) between the two groups. In the group of patients with BMI < 25 kg/m^2^, mOS was 67.7 months (95% CI 55.5–79.9 months), while for the patients with BMI ≥ 25 kg/m^2^, mOS was 64.6 months (95% CI 57.0–72.1 months, *P* = 0.990) (Figure 1).

**Figure 2:**
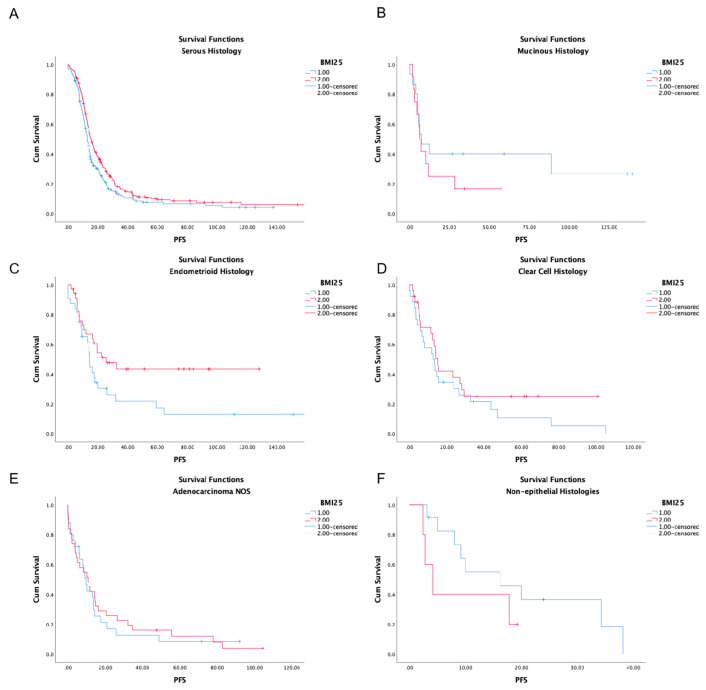
Kaplan–Meier curves of PFS according to BMI (BMI ≥ 25 kg/m^2^ (red line) and BMI < 25 kg/m^2^ (blue line) in different histological types (serous carcinoma (**A**), mucinous carcinoma (**B**), endometrioid carcinoma (**C**), clear cell carcinoma (**D**), adenocarcinoma not otherwise specified (**E**), non-epithelial carcinoma (**F**)).

To further elucidate the difference in mPFS between normal weight and overweight or obese patients, we examined PFS in different histological subtypes. The statistically significant benefit of overweight and obese patients was retained for patients with serous and endometrioid histology (mPFS 12.9 months, 95% CI 11.7–14.0 in patients with BMI < 25 kg/m^2^ vs. 15.6 months, 95% CI 13.9–17.3 in patients with BMI > 25 kg/m^2^, *P* = 0.012 and 14.6 months 95% CI 13.7–15.5 vs. 25.6 months, 95% CI 9.5–41.7, *P* = 0.031 respectively). No statistical difference was detected for patients, with mucinous, clear cell, adenocarcinoma not otherwise specified or non-epithelial histology (Figure 2 and Table 2).

**Figure 3:**
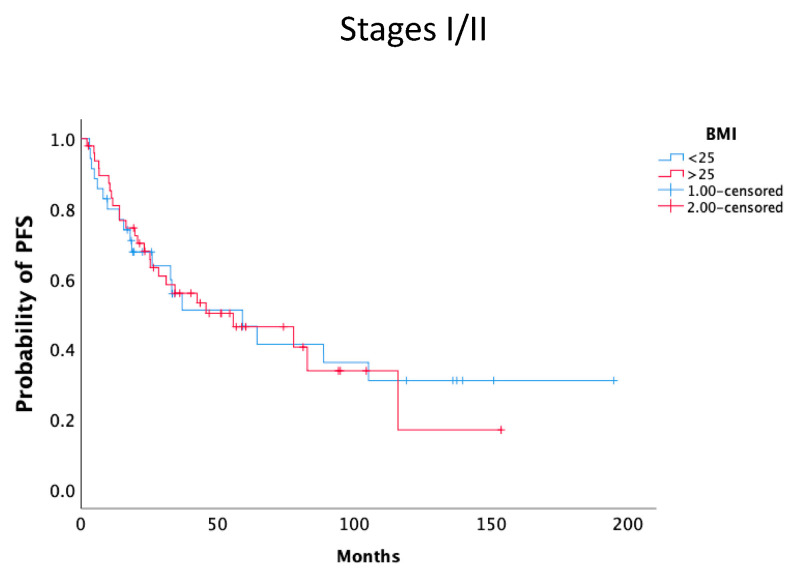
Kaplan–Meier curves of PFS according to BMI (BMI ≥ 25 kg/m^2^ (red line) and BMI < 25 kg/m^2^ (blue line) in disease stage I/II.

**Figure 4:**
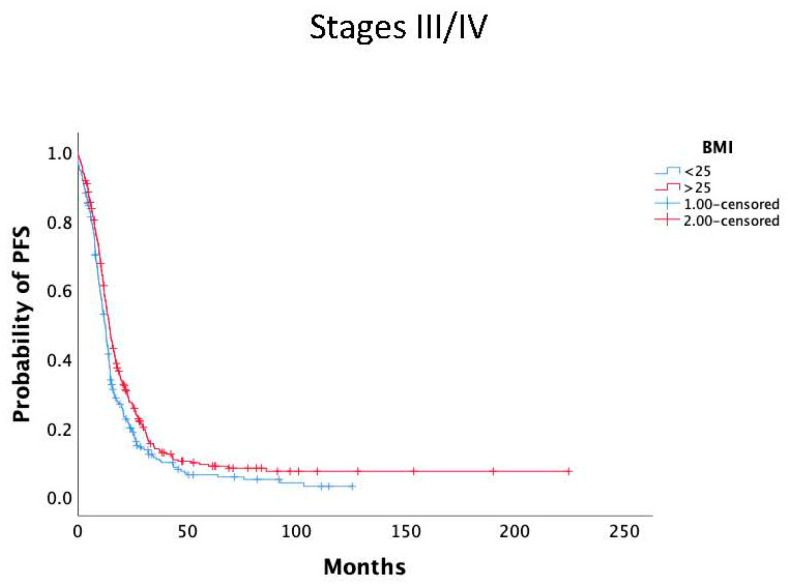
Kaplan–Meier curves of PFS according to BMI (BMI ≥ 25 kg/m^2^ (red line) and BMI < 25 kg/m^2^ (blue line) in disease stage III/IV.

We also analyzed the association of PFS with BMI in early stage (I/II) and advanced-stage (III/IV) disease. Again, patients with BMI ≥ 25 kg/m^2^ had statistically significantly better mPFS in comparison to those with BMI < 25 kg/m^2^ (14.2 months, 95% CI 12.9–15.6 vs. 12.3 months, 95% CI 11.2–13.4, *P* = 0.007). No statistically significant difference was noted in early stage patients who had a better overall prognosis (Figure 3 and Figure 4).

It is noteworthy that BMI < 25 kg/m^2^ was associated with increased relative risk for early recurrence of the disease (PFS < 6 months, relative risk 1.42, 95% CI 0.95–2.12) and platinum-resistant recurrence (platinum-free interval of less than 6 months, relative risk 1.68, 95% CI 1.21–2.33). The relative risk for early recurrence was analogous when standardized for age, and stage and was more prominent in patients with serous histology (RR 1.85, 95% CI 1.05–3.26).

**Figure 5:**
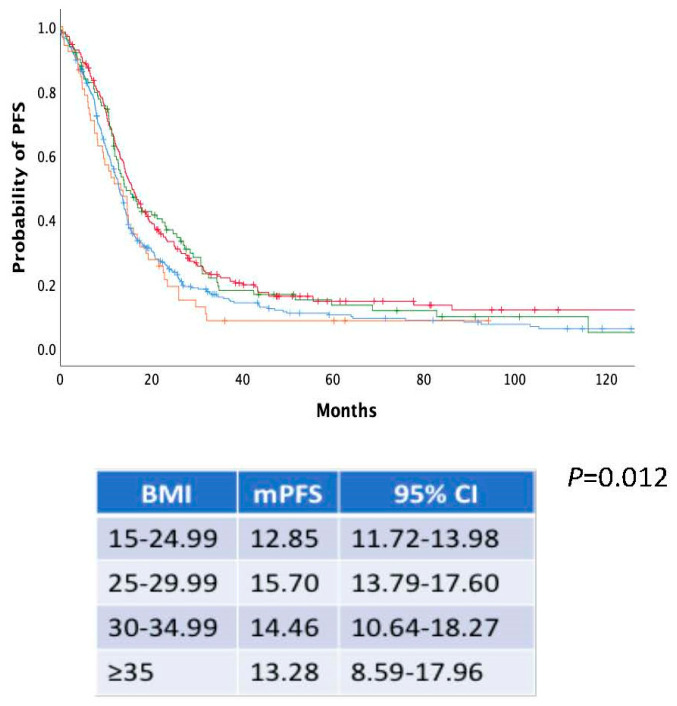
Kaplan–Meier curves of PFS according to BMI categorized as normal weight (BMI 15–24.99 kg/m^2^) (blue line), overweight (BMI 25–29.99 kg/m^2^) (red line), obese class I (BMI 30–34.99 kg/m^2^) (green line) and obese class II (BMI > 35 kg/m^2^) (orange line).

**Figure 6:**
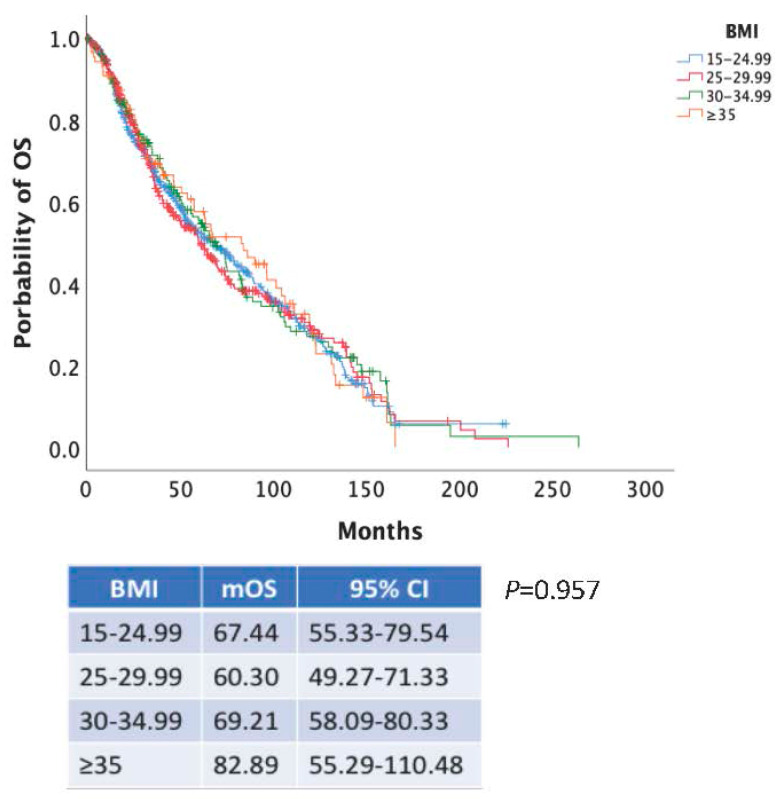
Kaplan–Meier curves of OS according to BMI categorized as normal weight (BMI 15–24.99 kg/m^2^) (blue line), overweight (BMI 25–29.99 kg/m^2^) (red line), obese class I (BMI 30–34.99 kg/m^2^) (green line) and obese class II (BMI > 35 kg/m^2^) (orange line).

We further investigated whether there was a linear trend between increased BMI and survival in the cohort. Thus, we categorized patients as normal weight (BMI 15–24.99 kg/m^2^), overweight (BMI 25–29.99 kg/m^2^), obese class I (BMI 30–34.99 kg/m^2^) and obese class II (BMI > 35 kg/m^2^). There was a statistically significant difference in mPFS between groups (mPFS: 12.85 vs. 15.70 vs. 14.46 vs. 13.28 months for the prespecified BMI groups, *P* = 0.012) (Figure 5). However, intergroup comparisons indicated that the survival benefit in comparison to normal weight patients was limited only to overweight patients (*P* = 0.005). The difference in PFS between patients with BMI > 35 kg/m^2^ and normal weight patients was not statistically significant. In contrast, no difference was noted for mOS (Figure 6).

## 4. Discussion

This study aimed to investigate the prognostic role of BMI before chemotherapy in the outcomes of women with ovarian cancer, focusing PFS and overall survival. Overall, our study indicated a statistically significant benefit in mPFS in overweight/obese patients (BMI ≥ 25 kg/m^2^) in comparison to normal weight (BMI < 25 kg/m^2^) ones. According to the subgroup analysis, this benefit was derived in patients with serous and endometrioid histology, but not for mucinous, clear cell, adenocarcinoma not otherwise specified, and non-epithelial histology. However, no significant difference was noted in median overall survival (mOS) between the two BMI groups.

Our findings are consistent with several studies suggesting an inclination toward improved survival in women with higher BMI [16,21]. In one specific study, while post-operative body weight did not significantly impact overall survival, a trend emerged indicating shorter progression-free survival in patients with a low or normal BMI compared to the obese subgroup. This trend may be linked to the potential of higher BMI patients to better tolerate chemotherapy, emphasizing the suggested association between low weight, suboptimal nutritional status, and potential effects on adjuvant therapy tolerance. Indeed, several lines of evidence indicate that lower BMI is associated with preterm discontinuation of treatment and an increased percentage of serious hematological toxicities in ovarian cancer patients [22]. This is also the case when antiangiogenetic treatment is also administered along with chemotherapy. In the OTILIA study that evaluated platinum-based chemotherapy plus bevacizumab in advanced ovarian cancer patients in clinical practice, tolerability of treatment was reduced in patients with BMI < 20 and more adverse events were noted [23]. In another study, preoperative prognostic nutritional index (PNI) was significantly associated with platinum resistance and survival [24]. Malnutrition and cachexia in patients with BMI < 20 kg/m^2^ create systemic inflammation and drive inflammation-induced platinum resistance [24]. The systemic inflammation induced in malnourished ovarian cancer patients with BMI < 20 kg/m^2^ and obese patients with BMI≥ 30 kg/m^2^ may influence cancer growth and metastasis and favor chemoresistance. This explains in part the results from the intergroup comparison that indicated a survival benefit only in overweight patients (BMI 25–29.99 kg/m^2^). Finally, our data may reflect in part the dose capping and the underdosing of carboplatin in obese patients [25,26]. Obese patients with BMI ≥ 30 kg/m^2^ are more likely to receive <85% of relative dose intensity (RDI) of carboplatin and this may impact PFS [26,27]. This is another reason why the survival benefit was limited to overweight patients and not expanded to obese patients as well.

It is also of interest that in our study patients with BMI ≥ 25 kg/m^2^ showed a statistically significant better mPFS in advanced-stage (III/IV) disease compared to those with BMI < 25 kg/m^2^. It is well established that advanced disease patients have worse prognoses, and the impact of obesity is clearly demonstrated in this subgroup of patients. Other studies involving advanced-stage epithelial ovarian cancer patients have also noted increased PFS for obese and overweight patients in comparison to normal weight ones, but no significant association in OS [21]. Notably, BMI < 25 kg/m^2^ was also associated with an increased relative risk for early recurrence of the disease and platinum-resistant recurrence. The findings highlight the complex relationship between BMI and ovarian cancer prognosis, suggesting that BMI may influence progression-free survival, particularly in certain histological subtypes and advanced-stage disease.

Surgical debulking and platinum-based chemotherapy remain the cornerstones in advanced ovarian cancer treatment. Despite the fact that obesity could be postulated to be an adverse factor for complete surgical removal of the disease, there was no difference in the outcome of debulking surgery between normal weight and overweight/obese patients in our study. Several lines of evidence in the literature indicate increased morbidity of ovarian cancer surgery among obese patients [28,29,30]. However, no difference was noted regarding post-surgery residual disease and the survival outcomes of these patients [29,30]. On the contrary, several studies have indicated underdosing of chemotherapy in obese ovarian cancer patients [26,31] is associated with adverse sequelae in the outcomes of these patients. This is specifically the case in the frontline treatment of the disease, despite the fact that there was no significant increase in chemotherapy-related adverse events in those patients who were treated according to their actual body weight [31]. In our study, all patients were treated on one site, and we applied chemotherapy according to the actual body weight of the patients. This has most probably functioned to unmask any effects of increased BMI in ovarian cancer survival, independent of treatment-related factors.

It is of interest also to examine our findings in the context of clinical and molecular heterogeneity of ovarian cancer. Molecular pathogenesis differs among the various histological types of epithelial ovarian cancer and this corresponds to differences both in overall prognosis and survival upon disease recurrence. The presence of genomic instability, mainly driven by mutations in BRCA1/2 as well as other genes of the homologous recombination repair pathway, determines pathogenesis in high-grade serous carcinomas and to a lesser extent high-grade endometrioid carcinomas [32]. These patients are known to have favorable mPFS and similar long-term OS in comparison to the non-BRCA mutant patients. Data evaluating the prognostic significance of BMI in these patients are sparse, which limits the formation of specific conclusions [33]. In addition, it has been recently demonstrated that obesity promotes carcinogenesis in BRCA1/2 mutation carriers through various pathways that induce DNA damage in epithelial cells [34], but there is no evidence for any adverse effects of obesity in the survival of BRCA mutant ovarian cancer patients. Therefore, molecular determinants of the disease are of utmost importance for the outcome of advanced ovarian patients, but do not seem to influence the survival benefit in overweight and obese patients with serous and endometrioid histology noted in our study.

On the other hand, evidence shows that BMI reduction during the course of or after chemotherapy is associated with poorer survival of ovarian cancer patients [18,19,20]. Changes in body weight during primary chemotherapy strongly correlate with overall survival. Weight loss during primary therapy signals poor OS, whereas weight gain serves as an indicator of improved survival [19]. In a study that conducted a comparison of PFS and OS across underweight, normal to overweight, and obese patients at diagnosis, post-surgery, and after treatment, findings revealed that only individuals with underweight status after treatment exhibited poorer OS compared to those with normal to overweight or obesity. Additionally, underweight patients experiencing a weight loss of ≥10% demonstrated inferior PFS and OS, in contrast to those with a weight loss of <10% [20]. This effect may be attributed to the capacity of individuals with obesity to withstand the elevated resting energy expenditure (REE) commonly associated with cancer. By enabling them to maintain their overall condition, a higher BMI potentially contributes to enhanced survival outcomes in this patient population [35].

Nevertheless, data on the association of BMI with progression-free survival (PFS) and overall survival (OS) in ovarian cancer patients remain conflicting. The lack of association between obesity as an independent factor and survival is supported by a number of studies that evaluated the weight either at diagnosis or at first chemotherapy [6,10,30,36]. Several studies have failed to find an association between pre-diagnosis BMI and either OS or PFS [8,9,10,11,12]. In a 2022 meta-analysis, Stelten et al. provided evidence against a significant association between BMI and overall survival, progression-free survival, disease-specific survival, or recurrence-free survival. Subgroup analyses based on BMI classifications (<30 kg/m^2^ and ≥30 kg/m^2^) failed to reveal statistically significant associations, highlighting the complexity of the relationship between BMI and EOC prognosis [15]. Interestingly, in one of these studies, when stratified by stage, BMI ≥ 35 was associated with lower survival in stages I/II and increased survival in stage IV patients [12]. In one study, the association between continuous post diagnosis BMI and lower ovarian cancer survival trended towards significance [37], while in a different study, post diagnosis BMI was associated with lower OS only for BMI ≥ 35 and with lower PFS for BMI = 30–35 [38]. Pavelka at al. found that a BMI > 25 at the time of treatment initiation was associated with shorter OS and PFS [13]. However, in a later study in which BMI was also assessed at the first chemotherapy, there was no statistically significant association between obesity and PFS or OS [14].

Results from the Ovarian Cancer Association Consortium (OCAC) indicate a 10–12% shorter OS and PFS in obese patients compared to those with normal weight. The OCAC included 21 studies that varied in the timing of BMI assessment from 5 years before to diagnosis date and the disadvantage to survival remained consistent in all measurement timepoints [5]. A 17% poorer survival in obese than in non-obese women has also been suggested in a 2012 meta-analysis of 14 studies. Although the patient’s BMI in the included studies was calculated at different time points, when stratified by the timing of the assessment a 13% shorter survival remained for all time groups (5 years prior to diagnosis, 1 year prior, and at the start of treatment) [39]. Lastly, a study on peritoneum-specific recurrence in EOC demonstrated the impact of BMI on outcomes. Patients with a high BMI experienced significantly shorter peritoneum-specific recurrence-free survival and overall survival compared to those with a normal BMI [40].

Concerning the effect of pre-diagnosis obesity, two studies found that a BMI ≥ 25 was associated with elevated ovarian cancer mortality [6,7], while a different study found a significant association only for BMI ≥ 35 [41]. In addition, obesity during early adulthood and remaining obese has also been associated with shorter ovarian cancer survival [9,37]. In another study, while premorbid obesity and overweight during early adulthood were identified as factors associated with a worse prognosis for patients with invasive EOC, there was no significant relationship between prognosis and obesity at the time of diagnosis, indicating that the timing of weight status might play a role in determining outcomes [42].

The discrepancies between results could possibly be explained by chemotherapy dose capping in obese individuals that occurred in certain cases in the first study but not in the latter [13,14]. It has been shown that women with a BMI ≥ 30 kg/m^2^ were more likely to be underdosed. This underdosing, especially in the neoadjuvant setting for serous tumors, was associated with inferior survival [26].

Body composition (BC) has shown more effective correlations than BMI and its correlation with survival. High adiposity and sarcopenia were associated with worse outcomes, adding a layer of complexity to the understanding of BC in the context of EOC prognosis [43]. In the meta-analysis of Stelten et al., while baseline BMI showed limited prognostic value, muscle mass emerged as a crucial determinant, with higher muscle mass being linked to improved progression-free survival. Additionally, higher muscle density was associated with better overall survival. The study emphasized the need for high-quality research with comprehensive reporting to better understand the implications of body composition measures for clinical outcomes [15].

The main strength of the current study is the large number of participants. This is one of the largest cohorts in the literature. All patients came from the same academic unit, which implies another strength, that all participants were followed up by the same multidisciplinary team and with the same protocol. Of course, this could also be considered as a limitation regarding the actual representation of the Greek population. However, our hospital is considered a center of excellence in the treatment of ovarian carcinoma, providing care to almost 15% of the women diagnosed annually with this neoplasm in the country. Numbers may be low for separate types of cancers and this can be considered as a limitation. Moreover, data were retrospectively analyzed, although they derived from a prospectively collected cohort. Also, patients with higher BMI were diagnosed at slightly earlier stages of I/II (23% versus 18% of those with BMI < 25 kg/m^2^). This may have affected our study results, although multivariate analysis was performed to include disease stage (Supplementary Table S2). Finally, in our study the definition of overweight or obesity is based only on BMI, as data on waist circumference (WC) or waist-to-hip ratio (WHR) were not available for the patients included in the study. Although BMI is still commonly used, its validity as a sole criterion for clinical obesity has been recently questioned in the literature and further markers have been proposed. Further prospective studies with more obesity markers will further elucidate the associations presented.

## 5. Conclusions

In conclusion, this study underscores the nuanced association between BMI and ovarian cancer prognosis. While a statistically significant difference in progression-free survival (PFS) was noted between normal weight and overweight/obese patients, with the latter group experiencing a survival benefit, no such difference was observed in overall survival (OS). Further exploration revealed the significant benefit to overweight and obese patients in certain histological subtypes, emphasizing the complexity of this relationship. Additionally, BMI ≥ 25 kg/m^2^ demonstrated a significant advantage in advanced-stage disease, with BMI < 25 kg/m^2^ correlating with an increased risk of early and platinum-resistant recurrence. Therefore, physicians dealing with such patients should be aware of the multifaceted impact of BMI on ovarian cancer outcomes, warranting comprehensive consideration in clinical contexts.

## Figures and Tables

**Table 1:** Clinicopathological characteristics of the total population and differences in their distribution among normal weight and overweight/obese patients.

Characteristic	Total Population	BMI < 25	BMI ≥ 25	
	**Median (25th–75th percentile)**	**Median (25th–75th percentile)**	**Median (25th–75th percentile)**	
**Age**	60.4 (51.2–68.6)	57.5 (49.0–67.5)	61.6 (52.2–69.1)	
**BMI**	25.5 (22.6–29.3)	22.4 (18.6–23.1)	28.7 (26.6–35.7)	
**BSA**	1.65 (1.55–1.76)	1.56 (1.49–1.63)	1.72 (1.65–1.82)	
	**N (%)**	**N (%)**	**N (%)**	***P***
**ECOG-PS**				0.454
0–1	905 (79.7%)	420 (87.5%)	482 (85.9%)	
≥2	139 (12.2%)	60 (12.5%)	79 (14.1%)	
Missing	92 (8.1%)	-	-	
**Stage**				0.023
I/II	228 (19.9%)	89 (17.7%)	139 (23.3%)	
III/IV	876 (77.3%)	414 (82.3%)	458 (76.7%)	
Missing	32 (2.8%)	-	-	
**Grade**				0.408
1	84 (7.4%)	42 (8.9%)	42 (7.5%)	
2	283 (24.9%)	121 (25.7%)	162 (29.0%)	
3	666 (58.6%)	305 (65.4%)	354 (63.4%)	
Missing	103 (9.1%)	-	-	
**Histology**				0.767
Serous	700 (61.6%)	315 (63.4%)	381 (63.4%)	
Mucinous	62 (5.5%)	30 (6.0%)	32 (5.3%)	
Endometrioid	137 (12.1%)	56 (11.3%)	81 (13.5%)	
Clear Cell	84 (7.4%)	41 (8.2%)	43 (7.2%)	
Adenocarcinoma NOS	93 (8.2%)	41 (8.2%)	52 (8.7%)	
Non-epithelial histologies	26 (2.3%)	14 (2.8%)	12 (2.0%)	
Missing	34 (3.0%)	-	-	
**Surgery**				0.623
PDS	978 (86.1)	450 (90.2%)	526 (88.6%)	
IDS	74 (6.5)	29 (5.8%)	43 (7.2%)	
No surgery	45 (4.0)	20 (4.0%)	25 (4.2%)	
Missing	39 (3.5%)	-	-	
**Surgical outcome**				0.376
Complete/Optimal	521 (45.9%)	231 (48.2%)	287 (51.0%)	
Suboptimal	524 (46.1%)	248 (51.3%)	276 (49.0%)	
Missing	91 (8.0%)	-	-	

**Table 2:** Median PFS according to BMI in different histological types (serous carcinoma, mucinous carcinoma, endometrioid carcinoma, clear cell carcinoma, adenocarcinoma not otherwise specified, non-epithelial carcinoma).

	Median PFS		
	BMI < 25	BMI ≥ 25	*P*
Serous	12.9 (11.7–14.0)	15.6 (13.9–17.3)	0.012
Mucinous	7.6 (0–16.0)	6.5 (4.3–8.6)	0.347
Endometrioid	14.6 (13.7–15.5)	25.6 (9.5–41.7)	0.031
Clear Cell	12.7 (5.6–19.7)	15.1 (12.5–17.6)	0.288
Adenocarcinoma NOS	9.6 (7.0–12.2)	10.7 (4.7–16.6)	0.759
Non-epithelial	16.2 (0.2–32.3)	4.2 (1.2–7.2)	0.193

## Data Availability

The data presented in this study are available on request from the corresponding author due to privacy reasons.

## Supplementary Materials

The following supporting information can be downloaded at: https://www.mdpi.com/article/10.3390/nu17030556/s1, Table S1: Clinicopathological characteristics of the subpopulation of patients according to BMI groups; Table S2: Multivariate analysis for PFS taking into consideration baseline prognostic clinicopathological parameters.

## Abbreviations

The following abbreviations are used in this manuscript:

BMI	Body Mass Index
EOC	Epithelial Ovarian Cancer
OCAC	Ovarian Cancer Association Consortium
OS	Overall Survival
PFS	Progression-Free Survival
PNI	Prognostic Nutritional Index
RDI	Relative Dose Intensity
REE	Resting Energy Expenditure
