# Supplementary material for: Possible Prognostic Role of BMI Before Chemotherapy in the Outcomes of Women with Ovarian Cancer
Source: Nutrients. 2025 Jan 31;17(3):556. doi: 10.3390/nu17030556 (PMC11820758; doi:10.3390/nu17030556)
Supplement: Supplementary file 1 [file nutrients-17-00556-s001.zip › nutrients-3449224-supplementary.pdf]

Supplementary Table S1. Multivariate analysis for PFS taking into consideration baseline prognostic clinicopathological parameters.

**Variable**	**HR (95% CI)**	***P***
**Age**		<0.001
<65	1	
≥65	1.54 (1.29-1.83)	
**Stage**		<0.001
I/II	1	
III/IV	3.37 (2.34-4.85)	
**ECOG-PS**		0.051
0-1	1	
2-4	1.30 (0.99-1.71)	
**Quality of Debulking surgery**		0.052
Optimal	1	
Suboptimal	1.19 (0.99-1.42)	
**BMI**		0.038
<25	1	
≥25	0.83 (0.70-0.99)	
**Histology**		0.266
Serous	1	
Mucinous	1.75 (1.04-2.94)	
Endometrioid	0.95 (0.67-1.34)	
Clear Cell	0.94 (0.67-1.33)	
Adenocarcinoma	1.21 (0.89-1.64)	

Non-epithelial	1.09 (0.59-2.02)	

ECOG-PS: Eastern Cooperative Oncology Group Performance Status, BMI: Body Mass Index

Supplementary Table S2. Clinicopathological characteristics of the subpopulation of patients according to BMI groups.

Characteristic	BMI 15-24.99 kg/m^2	BMI 25-29.99 kg/m^2	BMI 30-34.99 kg/m^2	BMI>35 kg/m^2	
	**Median (25th - 75th percentile)**	**Median (25th - 75th percentile)**	**Median (25th - 75th percentile)**	**Median (25th - 75th percentile)**	
**Age**	58.5 (49.3-67.8)	61.2 (52.3-70.0)	64.3 (56.9-71.4)	59.6 (51.8 – 67.4)	
**BMI**	22.4 (18.6-23.1)	26.9 (25.2-28.6)	31.6 (30.3-32.9)	37.8 (35.7-41.4)	
**BSA**	1.56 (1.49-1.63)	1.69 (1.61-1.77)	1.77 (1.69-1.85)	1.93 (1.86-2.07)	
	**N(%)**	**N(%)**	**N(%)**	**N(%)**	***P***
**ECOG-PS**					0.470
0-1	415 (87.4%)	300 (87.2%)	118 (84.3%)	64 (83.1%)	
≥2	60 (12.6%)	44 (12.8%)	22 (15.7%)	13 (16.9%)	
**Stage**					0.195
I/II	89 (17.8%)	84 (23.1%)	36 (23.5%)	19 (23.7%)	
III/IV	410 (82.2%)	280 (76.9%)	117 (76.5%)	61 (76.3%)	
**Grade**					0.517
1	42 (8.9%)	24 (6.9%)	15 (10.8%)	3 (4.1%)	
2	121 (25.7%)	103 (29.9%)	37 (26.6%)	22 (29.7%)	
3	305 (65.4%)	218 (63.2%)	87 (62.6%)	49 (66.2%)	
**Histology**					0.850
Serous	315 (63.4%)	239 (65.6%)	94 (60.3%	48 (55.3%)	
Mucinous	30 (6.0%)	21 (5.8%)	10 (6.4%)	1 (1.1%)	
Endometrioid	56 (11.3%)	44 (12.1%)	22 (14.1%)	15 (17.2%)	
Clear Cell	41 (8.2%)	24 (6.6%)	12 (7.7%)	7 (8.0%)	
Adenocarcinoma NOS	41 (8.2%)	31 (8.5%)	14 (8.9%)	7 (8.0%)	
Non-epithelial histologies	14 (2.8%)	5 (1.4%)	4 (2.6%)	3 (3.4%)	
**Surgery**					0.326
PDS	450 (90.2%)	314 (87.9%)	131 (86.2%)	81 (95.3%)	
IDS	29 (5.8%)	28 (7.8%)	12 (7.6%)	3 (3.5%)	
No surgery	20 (4.0%)	15 (4.2%)	9 (5.7%)	1 (1.2%)	
**Surgical outcome**					0.517
Complete/Optimal	230 (48.5%)	185 (53.2%)	67 (48.6%)	35 (45.5%)	
Suboptimal	244 (51.5%)	163 (46.8%)	71 (51.4%)	42 (54.5%)	

BMI: Body Mass Index, BSA: Body Surface Area, ECOG-PS: Eastern Cooperative Oncology Group Performance Status, PDS: Primary Debulking Surgery, IDS: Interval Debulking Surgery
